# Supplementary material for: Disordered clock protein interactions and charge blocks turn an hourglass into a persistent circadian oscillator
Source: Nat Commun. 2024 Apr 25;15:3523. doi: 10.1038/s41467-024-47761-z (PMC11045787; doi:10.1038/s41467-024-47761-z)
Supplement: Supplementary file 1 — Supplementary Information [file 41467_2024_47761_MOESM1_ESM.pdf]

Disordered clock protein interactions and charge blocks turn an hourglass into a persistent circadian oscillator.

Meaghan S. Jankowski, Daniel Griffith, Divya G. Shastry, Jacqueline F. Pelham, Garrett M. Ginell, Joshua Thomas, Pankaj Karande, Alex S. Holehouse, and Jennifer M. Hurley

Supplementary Figures

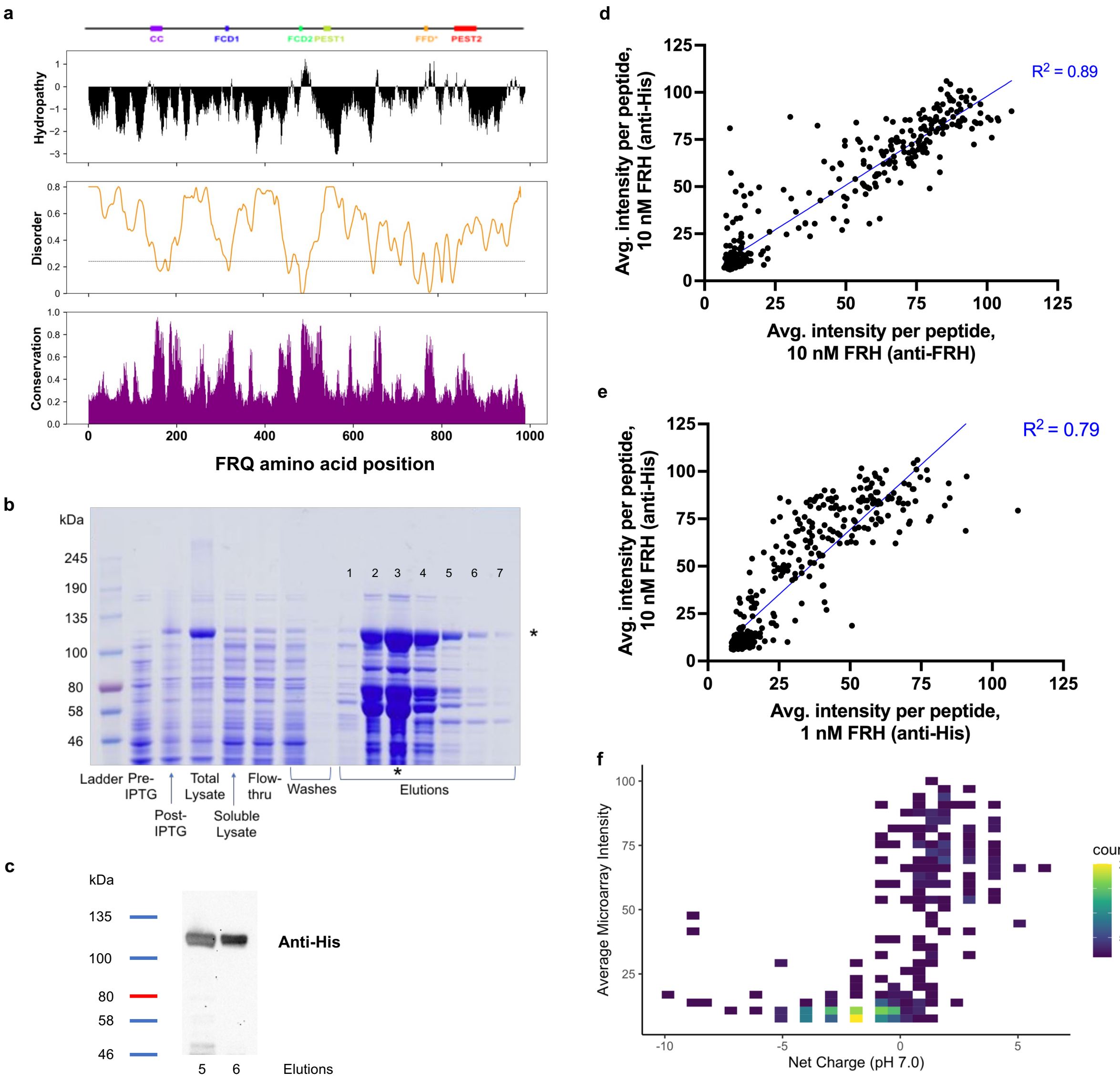

Supplementary Figure 1. FRQ is a highly disordered protein in the negative arm of the *N. crassa* clock that interacts with FRH. a, Linear sequence analysis of FRQ. Top: Linear hydropathy profile reveals per-residue hydrophobicity based on the Kyte-Doolittle scale [1]. The majority of the sequence is highly depleted for hydrophobic residues. Middle: Linear disorder profile calculated using Metapredict (v1), using a five-residue smoothing

window [2]. FRQ is predicted to be almost entirely disordered. Bottom: Per-residue conservation calculated across 83 FRQ orthologs taken from eggNOG [3]. Regions of high conservation coincide with hydrophobic regions with lower disorder tendencies. **b,** Coomassie-stained gel showing all steps of the FRHΔ100 purification, including pre- and post-IPTG addition and the total versus soluble lysate after French press and nickel column purification steps (flow-thru, washes and final elutions). The asterisk (*) denotes the elution used in subsequent microarray assays as well as the band that corresponds to FRHΔ100. **c,** Western blot of Nickel column elutions from (b), showing that the Anti-His antibody detects our full-length FRHΔ100 at ~115 kDa. **d,** Average normalized signal per peptide (average of n = 2-3 technical replicates) using anti-FRH to visualize binding vs. anti-His, using same ~10nM FRH concentration, shows a high Pearson correlation between these microarray results. **e,** Pearson correlation between experiments using two different concentrations of FRH (~1 nM versus ~10nM), show good reproducibility in average normalized signal per peptide (average of n = 2-3 technical replicates). Both (d) and (e) are results from Library 1 peptides. **f,** Densitometry plot of data shown in Fig. 2c, made using R. Source data are provided as a Source Data file and at [https://zenodo.org/records/10793684]. Related to Figs. 1 and 2.

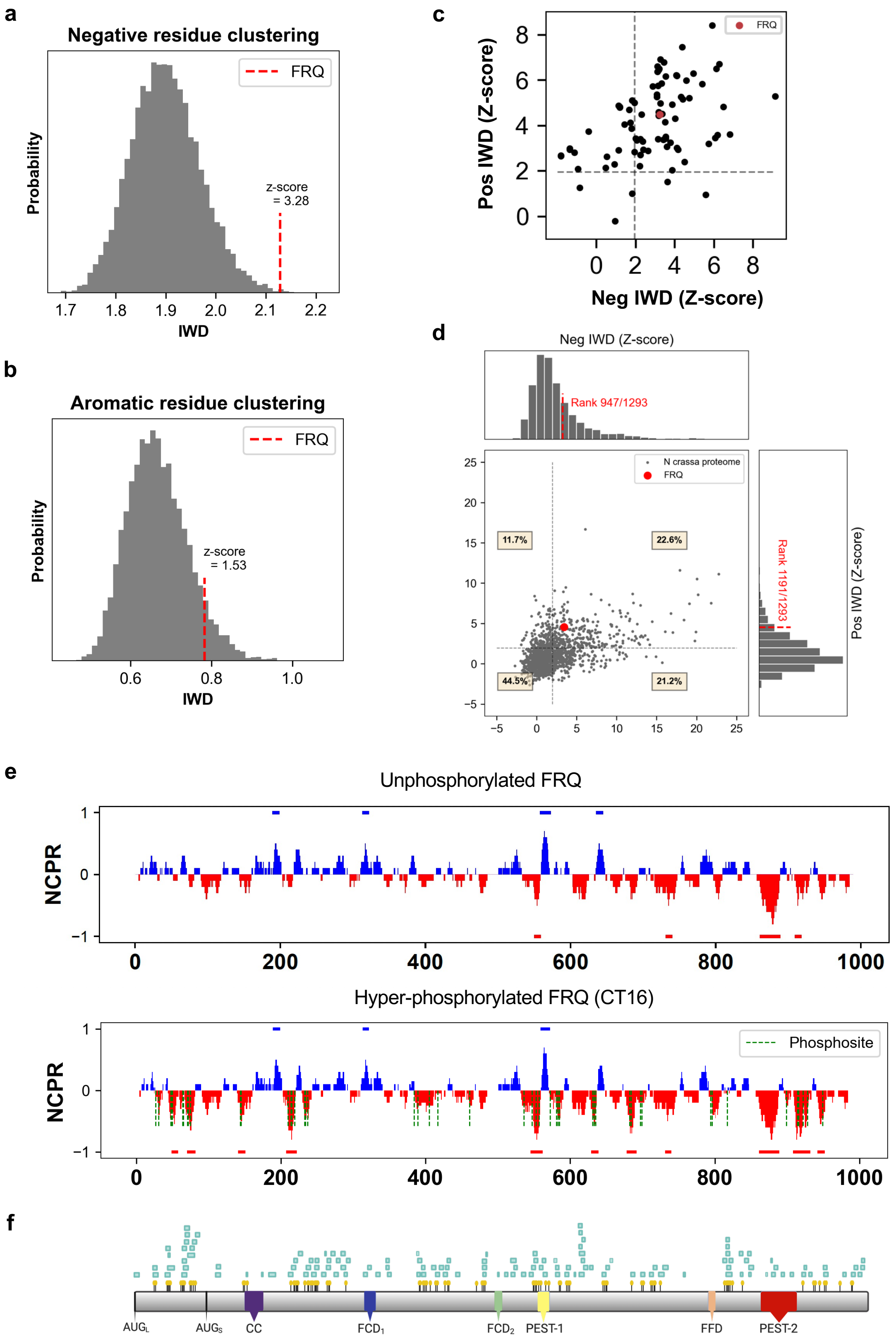

a
Negative residue clustering
FRQ
z-score
= 3.28
Probability
IWD
b
Aromatic residue clustering
FRQ
z-score
= 1.53
Probability
IWD
c
FRQ
Pos IWD (Z-score)
Neg IWD (Z-score)
d
Neg IWD (Z-score)
Rank 947/1293
N crassa proteome
FRQ
11.7%
22.6%
44.5%
21.2%
Rank 1191/1293
Pos IWD (Z-score)
e
Unphosphorylated FRQ
NCPR
Hyper-phosphorylated FRQ (CT16)
Phosphosite
NCPR
f
AUGL
AUGS
CC
FCD1
FCD2
PEST-1
FFD
PEST-2

Supplementary Figure 2. FRQ is highly ranked within the *N. crassa* proteome for having positively charged clusters while SLiMs tend to occur in segments that become negatively charged with increasing FRQ phosphorylation. a, The statistical significance of negatively charged clusters of residues is assessed by calculating the expected null distribution for negatively charged residue clustering from randomly shuffled sequences that match the composition and length of FRQ. Clustering is calculated using the Inverse Weighted Distance (IWD) (see Methods). The WT FRQ sequence (red dashed line) has negatively charged residues that are more well clustered than almost all randomly shuffled sequences. **b,** The WT FRQ sequence (red dashed line) does not have significant clustering of aromatic residues relative to randomly shuffled FRQ sequences (Z-score > 1.96 is $p < 0.05$). **c,** The Z-score of positive residue Inverse Weighted Distance (Pos IWD Z-score) versus negative residue IWD (Neg IWD Z-score) of FRQ (red circle) compared to 86 FRQ orthologs (black circles). Dotted lines denote significance (Z-score > 1.96 is $p < 0.05$). Most sequences (64/86) show significant clustering of both positively charged and negatively charged residues (top right quadrant). A subset of sequences (17/86) only show clustering of positively charged residues (top left quadrant). **d,** A comparison of FRQ to 1293 randomly selected *N. crassa* proteins in terms of Pos IWD Z-score and Neg IWD Z-score, showing that FRQ (red dot) is in the top 7% of these proteins in terms of how clustered its positive charge residues are. The dotted lines denote significance (Z-score > 1.96 is $p < 0.05$). **e,** Net Charge Per Residue (NCPR) plot of FRQ using a scanning window of 10 a.a., comparing no phosphorylation versus all known phosphorylated residues at CT16 (phosphosite positions shown with dotted green lines), as published in [4]. Blue lines above the NCPR plot denote positive charge blocks (NCPR >= 0.5 for at least one 10 a.a. segment in a charge block [5]) while red lines below denote negative charge blocks (NCPR <= -0.5). CT = Circadian Time (0 is relative dawn and 12 is relative dusk). Note that the unphosphorylated FRQ NCPR plot is also shown in Fig. 3a. **f,** Predicted SLiMs (green boxes) from the ELM database for verified interactors of FRQ, along with detected phosphosites denoted with yellow pins [4,6,7]. See Fig. 1 for more details about the known FRQ domains highlighted in color. (**f**) made with Biorender. Source data are provided as a Source Data file and at [https://zenodo.org/records/10793684]. Related to Fig. 3.

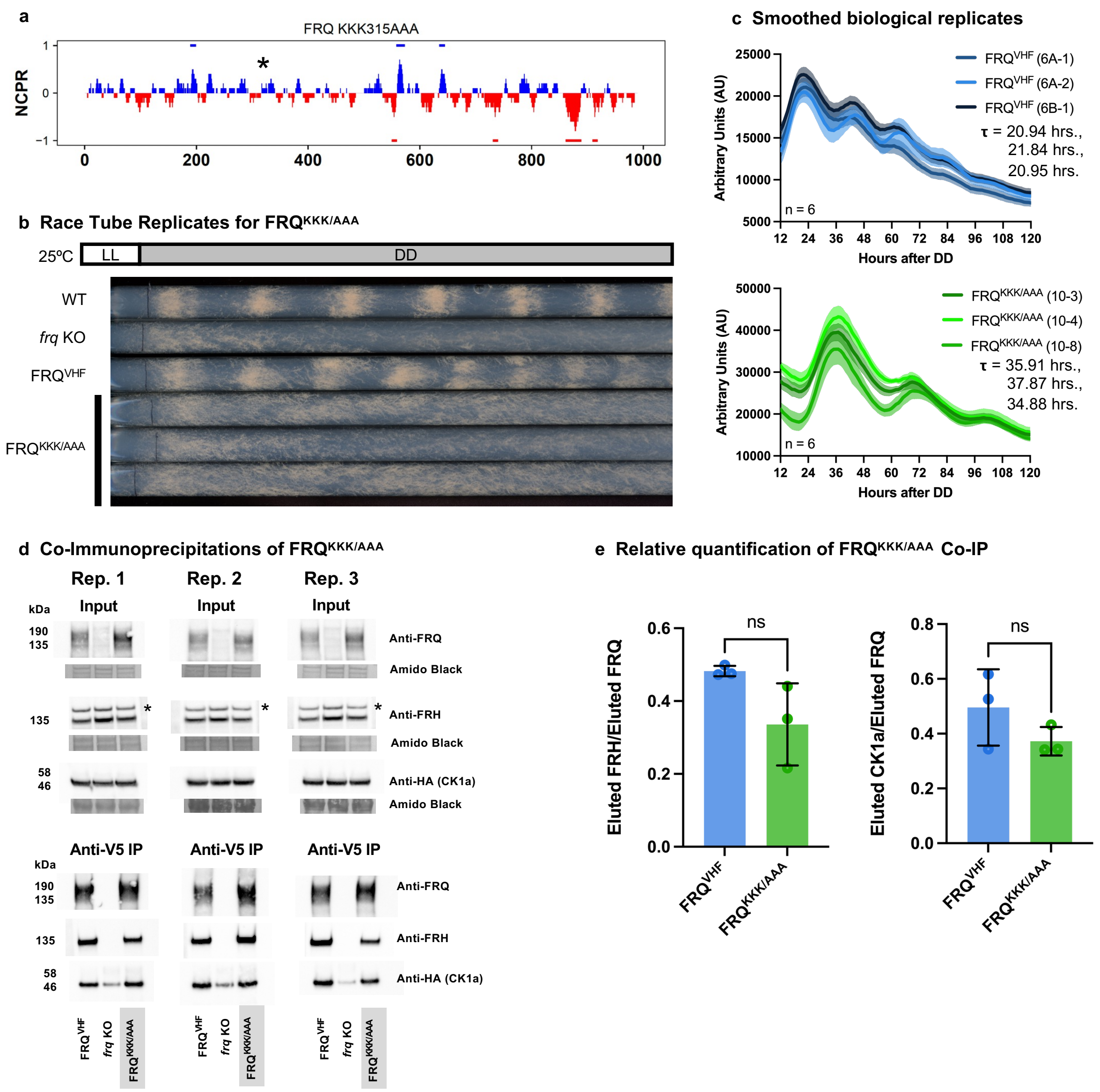

Supplementary Figure 3. Ablating a positive charge block leads to loss of rhythmic output and a lengthening of core clock period but no significant change in binding to FRH or CK1A. a, Net Charge Per Residue (NCPR) plot showing the location of the `KKK315AAA` mutation (asterisk) and the loss of the positive charge block in that location when compared to Fig. 3a. Blue lines at top of NCPR plot denote positive charge blocks while red lines below plot denote negative charge blocks. Sliding window was 10 a.a. Related to Fig. 3. **b,** Race tube replicates of the $FRQ^{KKK/AAA}$ mutant strain. **c,** Smoothed luciferase traces (n = 6) from FRQ^{VHF} and $FRQ^{KKK/AAA}$ transformants. Fitted periods (τ) are from ECHO (v3.22). **d,** Western blots of the anti-Flag co-immunoprecipitation of the described VHF-tagged FRQ strains to investigate changes in FRQ binding to both FRH and CK-1A. Note some residual CK-1A binding to the anti-flag beads in our *frq* KO strain, but the $FRQ^{KKK/AAA}$ strain shows more and similar amounts of CK-1A as was bound to our wild-type FRQ^{VHF}. **e,** Relative quantification (Image Lab v6.0) of Co-IPs shown in (**d**), with

the Adjusted Total Band Volume of eluted FRH (on the left) or eluted CK1A:HA (on the right) divided by the Adjusted Total Band Volume of eluted FRQ for FRQ^{VHF} versus $FRQ^{KKK/AAA}$. Neither showed significant changes based on a non-parametric two-tailed Mann Whitney test ($p = 0.1$ for FRH/FRQ and $p = 0.2$ for CK1A/FRQ, ns = not significant). Source data are provided as a Source Data file, at [https://doi.org/10.17632/7hgspb5gn7.1] and [https://zenodo.org/records/10793684]. Related to Fig. 3.

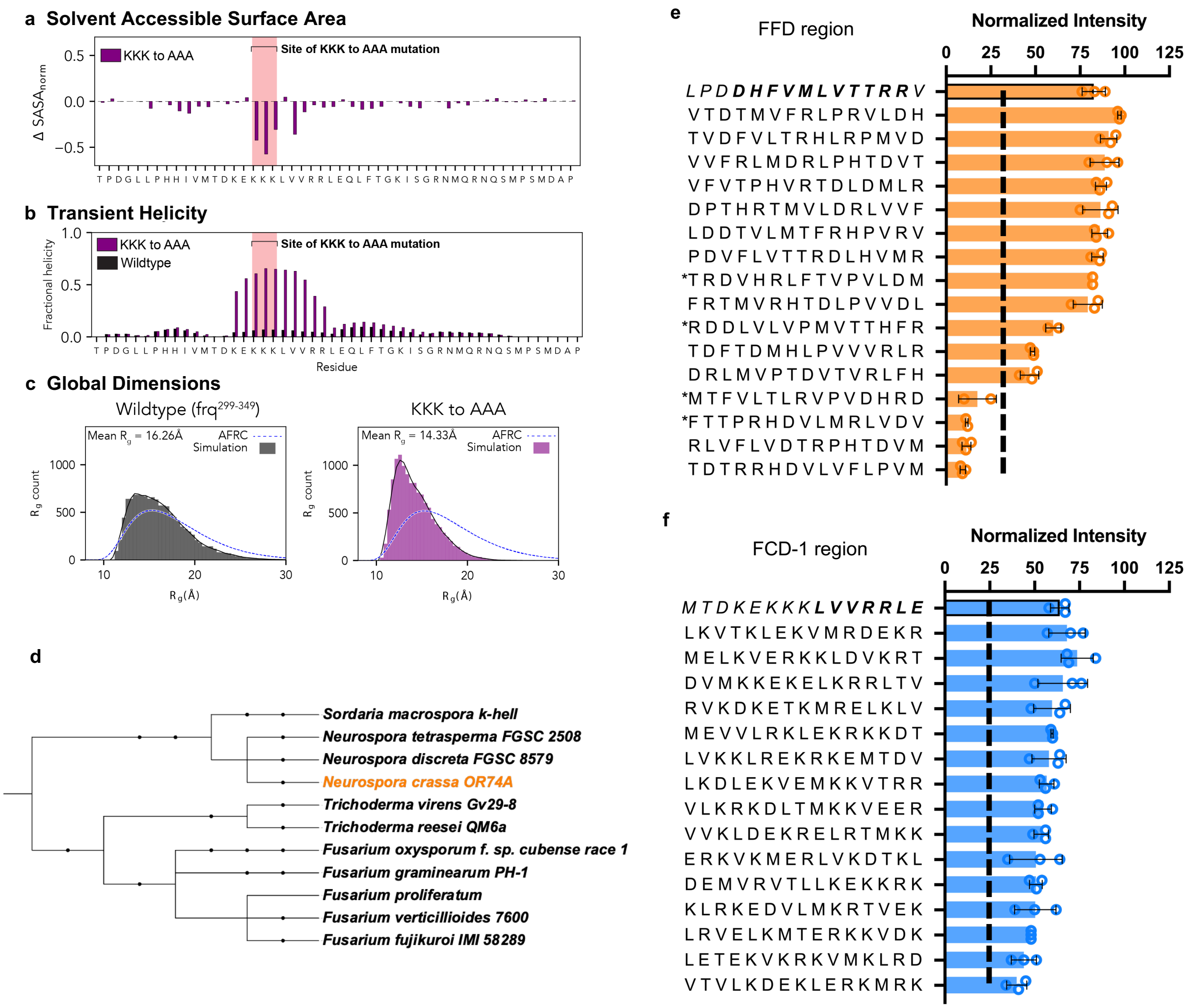

Supplementary Figure 4. All-atom Monte Carlo suggests no significant changes to FCD-1 availability when mutated while sequence composition contributes to the interaction between the FRQ-FFD and FRH. Results from an all-atom Monte Carlo simulation of a 50-residue portion of FRQ centered on the FCD-1 region (see Methods), showing changes in **(a)** the normalized solvent accessible surface area (SASA) for each residue along with **(b)** transient helicity. **c,** The global dimensions of the simulated ensembles are assessed based on histograms of the radius of gyration (R_g), a measure of overall ensemble size. AFRC = Analytical Flory Random Coil (see Methods). **d,** A phylogenetic tree of the FRQ orthologs considered in the FFD region alignment shown in Fig. 4a. **e,** Normalized binding intensity of the native and scrambled peptides of the FRQ-FFD region to FRH, based on peptides from Library I, using ~10 nM FRH and visualized using anti-His. Peptide sequences are reported along the y-axis with the native FFD in bold. Normalized binding intensity values are reported for each peptide along the x-axis and error bars report the standard deviation (n = 3 technical replicates, except n = 2 for peptides marked with *). The dashed line represents one Standard Deviation above background. **f,** Normalized binding intensity of the native and scrambled peptides of the FRQ-FCD-1 region to FRH, based on peptides from Library II, using ~100 nM FRH and visualized using anti-His. Peptide sequences are reported along the y-axis with the native

FCD-1 in bold. Binding intensity values are reported for each peptide along the x-axis, and error bars report the standard deviation (n = 3 technical replicates). The dashed line represents one standard deviation above background-subtracted zero, for the whole library. Source data are provided as a Source Data file, at [https://doi.org/10.17632/7hgspb5gn7.1] and [https://zenodo.org/records/10793684]. Related to Fig. 4.

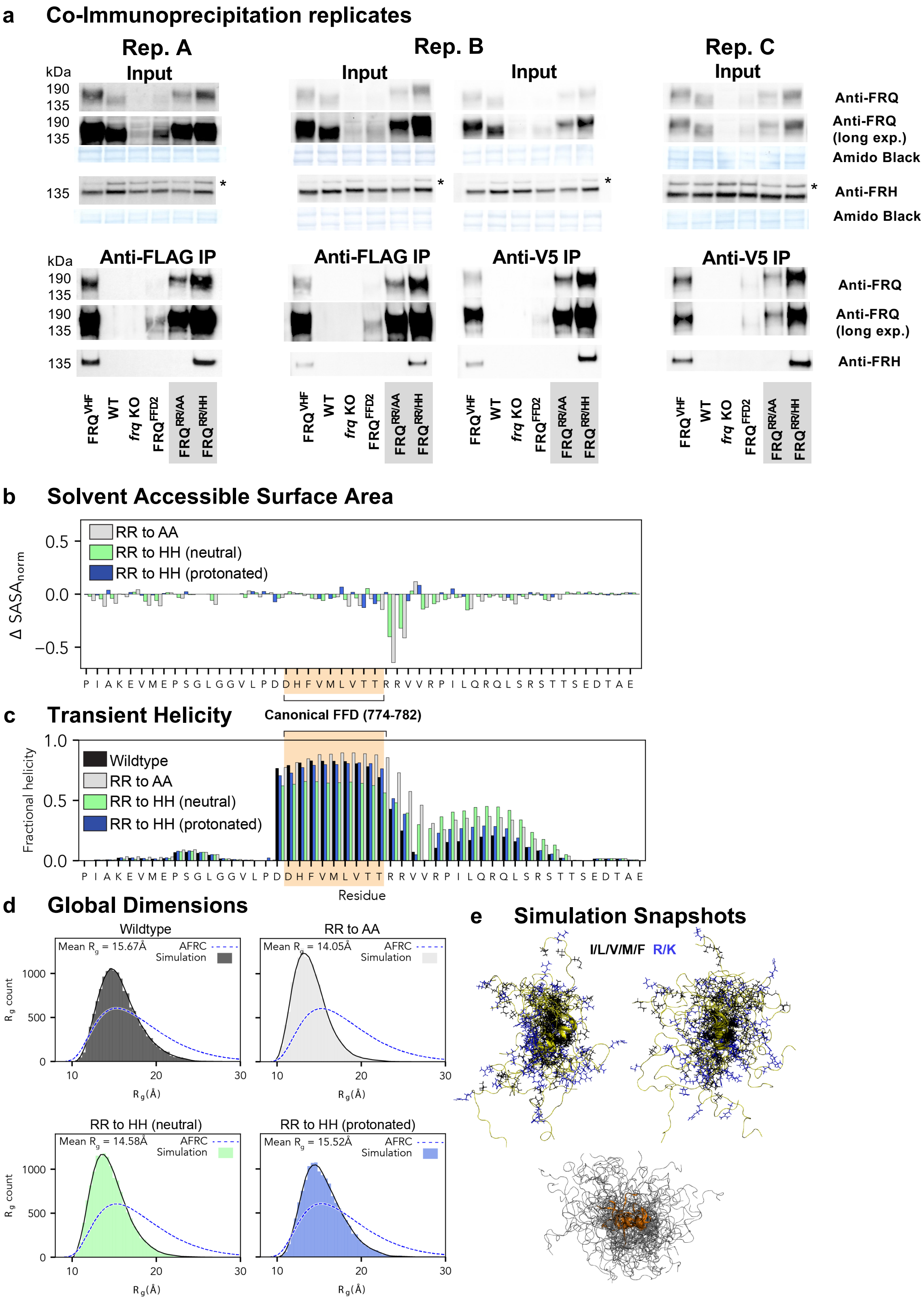
a Co-Immunoprecipitation replicates
Rep. A
Rep. B
Rep. C
Input
kDa
190
135
Anti-FRQ
Anti-FRQ (long exp.)
Amido Black
Anti-FRH
Anti-FLAG IP
Anti-V5 IP
FRQ^VHF
WT
frq KO
FRQ^FFD2
FRQ^RR/AA
FRQ^RR/HH
b Solvent Accessible Surface Area
Δ SASAnorm
RR to AA
RR to HH (neutral)
RR to HH (protonated)
P I A K E V M E P S G L G G V L P D D H F V M L V T T R R V V R P I L Q R Q L S R S T T S E D T A E
Canonical FFD (774-782)
c Transient Helicity
Fractional helicity
Wildtype
RR to AA
RR to HH (neutral)
RR to HH (protonated)
Residue
d Global Dimensions
Wildtype
Mean Rg = 15.67Å
RR to AA
Mean Rg = 14.05Å
RR to HH (neutral)
Mean Rg = 14.58Å
RR to HH (protonated)
Mean Rg = 15.52Å
AFRC
Simulation
Rg count
Rg(Å)
e Simulation Snapshots
I/L/V/M/F R/K

Supplementary Figure 5. Loss of essential binding residues leads to loss of FRH interaction without a change in the FRQ FFD microenvironment. a, Biological replicates of the Input and anti-FLAG immunoprecipitation (IP) or anti-V5 IP for the noted strains. Note that Replicate B shows a technical replicate from carrying out anti-FLAG and anti-V5 IPs on the same harvested tissue. Asterisk (*) denotes a non-specific band in the lysate lanes when using anti-FRH. **b,** The change in normalized solvent accessible surface area (SASA) for each residue in a 50-residue FFD region assessed by all-atom Monte Carlo simulations (see Methods). Other than the arginine residues themselves, no significant changes in solvent accessibility are observed upon changing the essential arginine residues to alanine, neutral histidine, or protonated histidine. The canonical FFD region is highlighted in orange. **c,** Transient helicity reveals the canonical FFD lies along a region that is predominantly in a transient helix, a common binding mode for intrinsically disordered regions. `RR783` mutations do not alter helicity profiles, with the exception of `RR783AA` which becomes slightly more helical. **d,** Global dimensions of the four ensembles are assessed based on histograms of the radius of gyration (R_g), a measure of overall ensemble size compared to AFRC (Analytical Flory Random Coil, see Methods). All four sequences have almost identical global dimensions. **e,** Snapshots of superimposed structures from a subset of the simulations also shown in Supplementary Movie 1. Positively charged residues are shown in blue, hydrophobic residues in black, and the chain backbone in yellow. On the bottom, helical conformations are shown in orange, illustrating the transient and highly disordered nature of the ensemble. Source data are provided as a Source Data file and at [https://zenodo.org/records/10793684]. Related to Figs. 4 and 5.

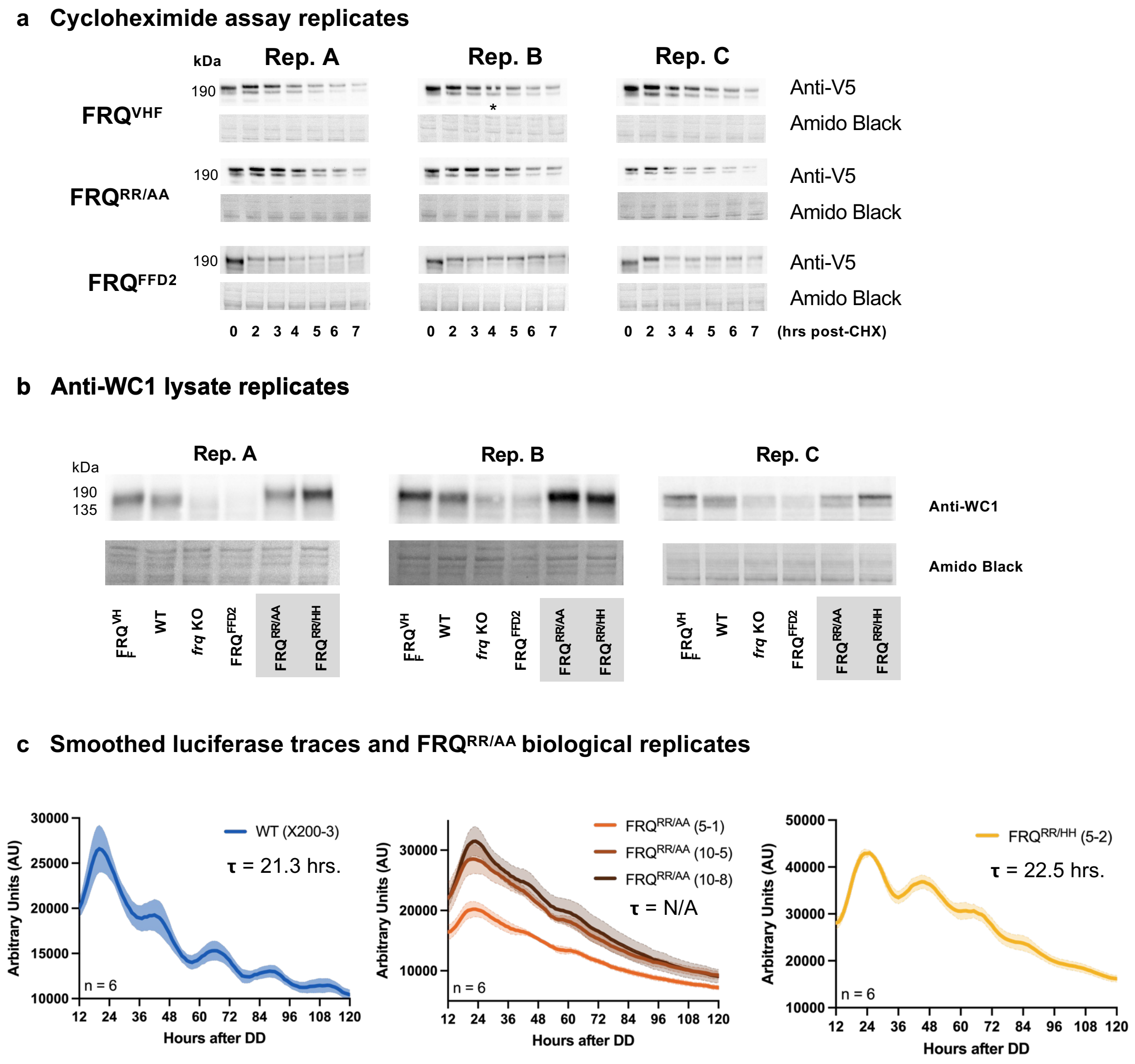

Supplementary Figure 6. Stable FRQ$^{RR/AA}$ supports wild-type levels of WC-1, suggesting the closure of the TTFL independent of FRH binding. a, Biological replicate blots and Amido Black stained membranes for the cycloheximide assay. Asterisk (*) notes that this lane was omitted from analysis due to an air bubble. **b,** Replicates of anti-WC-1 lysate levels amongst the different strains, and matching Amido Black stained membranes showing even loading. Note that replicates A and B were run on Tris-Acetate gels (3-8%) while replicate C was run on a Bis-Tris gel (4-12%). **c,** Smoothed Luciferase Traces (n = 6) for each of the indicated strains, measured in Arbitrary Units (AU). Fitted periods (τ) are from ECHO (v3.22). Source data are provided as a Source Data file and at [https://doi.org/10.17632/7hgspb5gn7.1]. Related to Figs. 5 and 6.

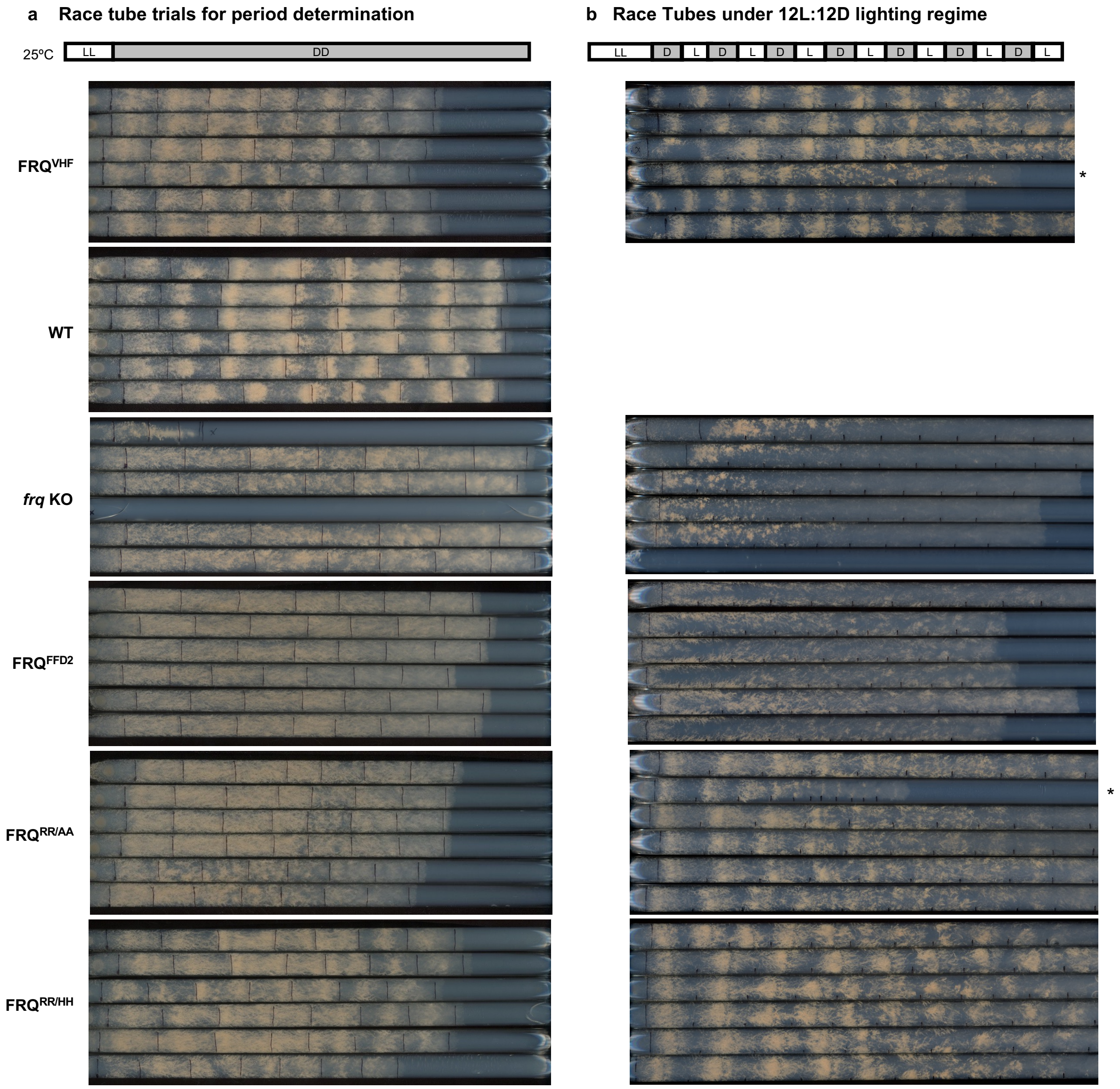

Supplementary Figure 7. FRQ$^{RR/AA}$ is overtly arrhythmic in constant conditions yet rhythmic in a 12L:12D lighting regime. a, Race tubes grown in constant light (LL) before being allowed to free run in constant dark (DD) for each of the described *N. crassa* strains. **b,** Race tubes grown in a 12 hr. light: 12 hr. dark (12L:12D) lighting regime for each of the described *N. crassa* strains. Daily marks are denoted with black lines. Asterisk (*) denotes that these race tubes were omitted from period analysis due to insufficient number of bands and/or poor growth. Note that arrhythmic strains that had no daily banding were not considered for period analysis. Source data are provided as a Source Data file. Related to Fig. 6.

Supplementary References

1. Kyte, J. & Doolittle, R. F. A simple method for displaying the hydropathic character of a protein. *J Mol Biol* **157**, 105–132 (1982).
2. Emenecker, R. J., Griffith, D. & Holehouse, A. S. Metapredict: a fast, accurate, and easy-to-use predictor of consensus disorder and structure. *Biophysical Journal* **120**, 4312–4319 (2021).
3. Huerta-Cepas, J. *et al.* eggNOG 5.0: a hierarchical, functionally and phylogenetically annotated orthology resource based on 5090 organisms and 2502 viruses. *Nucleic Acids Research* **47**, D309–D314 (2019).
4. Baker, C. L., Kettenbach, A. N., Loros, J. J., Gerber, S. A. & Dunlap, J. C. Quantitative Proteomics Reveals a Dynamic Interactome and Phase-Specific Phosphorylation in the Neurospora Circadian Clock. *Molec. Cell* **34**, 354–363 (2009).
5. Lyons, H. *et al.* Functional partitioning of transcriptional regulators by patterned charge blocks. *Cell* **186**, 327–345 (2023).
6. Gouw, M. *et al.* The eukaryotic linear motif resource – 2018 update. *Nucleic Acids Research* **46**, D428–D434 (2017).
7. Pelham, J. F. *et al.* Conformational changes in the negative arm of the circadian clock correlate with dynamic interactomes involved in post-transcriptional regulation. *Cell Rep.* **42**, 112376 (2023).
