# Supplementary material for: Disordered clock protein interactions and charge blocks turn an hourglass into a persistent circadian oscillator
Source: Nat Commun. 2024 Apr 25;15:3523. doi: 10.1038/s41467-024-47761-z (PMC11045787; doi:10.1038/s41467-024-47761-z)
Supplement: Supplementary file 3 — Description of Additional Supplementary Files [file 41467_2024_47761_MOESM3_ESM.pdf]

Description of Additional Supplementary Files:

Supplementary Movie 1: Movie of Monte Carlo all-atom simulation showing the overall disordered nature, albeit with transient helices, that characterizes the FFD region of FRQ. Residues 754-803 of Wild-type FRQ were simulated using the programs CAMPARI and ABSINTH (see Methods for complete details). Residue and helices colored as in Supplementary Fig. 5e.
