# Supplementary material for: Disordered clock protein interactions and charge blocks turn an hourglass into a persistent circadian oscillator
Source: Nat Commun. 2024 Apr 25;15:3523. doi: 10.1038/s41467-024-47761-z (PMC11045787; doi:10.1038/s41467-024-47761-z)
Supplement: Supplementary file 5 — Reporting Summary [file 41467_2024_47761_MOESM5_ESM.pdf]

nature portfolio

Corresponding author(s): Jennifer M Hurley

Last updated by author(s): Apr 10, 2024

Reporting Summary

Nature Portfolio wishes to improve the reproducibility of the work that we publish. This form provides structure for consistency and transparency in reporting. For further information on Nature Portfolio policies, see our Editorial Policies and the Editorial Policy Checklist.

Statistics

For all statistical analyses, confirm that the following items are present in the figure legend, table legend, main text, or Methods section.

n/a	Confirmed	
☐	☒	The exact sample size (*n*) for each experimental group/condition, given as a discrete number and unit of measurement
☐	☒	A statement on whether measurements were taken from distinct samples or whether the same sample was measured repeatedly
☐	☒	The statistical test(s) used AND whether they are one- or two-sided<br>*Only common tests should be described solely by name; describe more complex techniques in the Methods section.*
☒	☐	A description of all covariates tested
☐	☒	A description of any assumptions or corrections, such as tests of normality and adjustment for multiple comparisons
☐	☒	A full description of the statistical parameters including central tendency (e.g. means) or other basic estimates (e.g. regression coefficient) AND variation (e.g. standard deviation) or associated estimates of uncertainty (e.g. confidence intervals)
☐	☒	For null hypothesis testing, the test statistic (e.g. F, t, r) with confidence intervals, effect sizes, degrees of freedom and P value noted<br>*Give P values as exact values whenever suitable.*
☒	☐	For Bayesian analysis, information on the choice of priors and Markov chain Monte Carlo settings
☒	☐	For hierarchical and complex designs, identification of the appropriate level for tests and full reporting of outcomes
☒	☐	Estimates of effect sizes (e.g. Cohen's *d*, Pearson's *r*), indicating how they were calculated

Our web collection on statistics for biologists contains articles on many of the points above.

Software and code

Policy information about availability of computer code

Data collection	Image Lab Software v6.0.1, AlphaFold v2.1.0, Lightfield v5.2, CAMPARI with ABSINTH v3, FungiDB release 47
Data analysis	Image Lab Software v4.0 and v6.0.1, TIGR Spotfinder (Release 2009-08-01), Prism v9.0.2, ECHO v3.22, FIJI v2.0.0, Custom ImageJ code "Toolset Image Analysis Larrondo's Lab 1.0", ChronOSX v1.1.0, SOURSOP v0.2.0, localCIDER v0.1.18, EGGNOG v5.0, metapredict v1, SnapGene v5.1.1, Skylign (accessed July 2020), Interactive Tree of Life v.4, PyMol v2.4.0, R v4.3.1.

For manuscripts utilizing custom algorithms or software that are central to the research but not yet described in published literature, software must be made available to editors and reviewers. We strongly encourage code deposition in a community repository (e.g. GitHub). See the Nature Portfolio guidelines for submitting code & software for further information.

Data

Policy information about availability of data

All manuscripts must include a data availability statement. This statement should provide the following information, where applicable:

- Accession codes, unique identifiers, or web links for publicly available datasets
- A description of any restrictions on data availability
- For clinical datasets or third party data, please ensure that the statement adheres to our policy

The authors declare that the data supporting the findings of this study are available within the paper and its supplementary materials. The designed FRQ peptide libraries, original FRQ peptide microarray images, extracted peptide microarray intensity values, luciferase reporter CCD array trials, and relative luminescence

values are included in Mendeley Datasets (https://doi.org/10.17632/7hgspb5gn7.1). All associated inputs, subsampled trajectories and analyses related to the All-Atom Monte Carlo simulations are available on Zenodo (https://zenodo.org/records/10793684). Additionally, a previously solved crystal structure of FRH that appears in Figs. 2d and 6e can be found in the PDB under accession code 5E02 [https://doi.org/10.2210/pdb5e02/pdb]. Source data are included with this paper.

Research involving human participants, their data, or biological material

Policy information about studies with human participants or human data. See also policy information about sex, gender (identity/presentation), and sexual orientation and race, ethnicity and racism.

Reporting on sex and gender	n/a
Reporting on race, ethnicity, or other socially relevant groupings	n/a
Population characteristics	n/a
Recruitment	n/a
Ethics oversight	n/a

Note that full information on the approval of the study protocol must also be provided in the manuscript.

Field-specific reporting

Please select the one below that is the best fit for your research. If you are not sure, read the appropriate sections before making your selection.

☒ Life sciences ☐ Behavioural & social sciences ☐ Ecological, evolutionary & environmental sciences

For a reference copy of the document with all sections, see nature.com/documents/nr-reporting-summary-flat.pdf

Life sciences study design

All studies must disclose on these points even when the disclosure is negative.

Sample size	No sample size calculations were performed. Standard triplicate technical replicates were used in the peptide microarray assays, and repeated with different antibodies and protein concentrations. For in-vivo work, biological triplicates are typical in our field.
Data exclusions	This is described in the methods section, but briefly, for the peptide microarrays there can be printing errors on the slides causing merged or missing/misshapen spots, with these spots excluded from further analysis. Peptides that had less than two technical replicates were also excluded from downstream analyses. In race tube experiments, tubes that had Neurospora that did not grow or stopped growing before the end of the trial were excluded from period analysis. Arrhythmic strains were not analyzed for periods, as there were no daily bands to detect. For western blot relative quantifications, protein bands with air bubbles were excluded from analysis. For camera trials following pfrq:luciferase, wells with less media, low growth, or low/lost signal during the trial were excluded from further analyses.
Replication	For in vitro work, comparing our results using different antibodies and different protein concentrations for our peptide microarrays allowed us to show that results were replicable. In addition to biological replicates of n = 3 to ensure replicability, additional transformants were also used to ensure any unexpected findings were not due to any off-target transformation effects (e.g. three transformants for pfrq:luc strains for FRQ(KKK/AAA) and FRQ(RR/AA)).
Randomization	There were no experimental treatment groups, therefore no randomization was carried out.
Blinding	There were no experimental treatment groups, therefore no blinding was done.

Reporting for specific materials, systems and methods

We require information from authors about some types of materials, experimental systems and methods used in many studies. Here, indicate whether each material, system or method listed is relevant to your study. If you are not sure if a list item applies to your research, read the appropriate section before selecting a response.

Materials & experimental systems

n/a	Involved in the study
☐	☒ Antibodies
☒	☐ Eukaryotic cell lines
☒	☐ Palaeontology and archaeology
☒	☐ Animals and other organisms
☒	☐ Clinical data
☒	☐ Dual use research of concern
☒	☐ Plants

Methods

n/a	Involved in the study
☒	☐ ChIP-seq
☒	☐ Flow cytometry
☒	☐ MRI-based neuroimaging

Antibodies

Antibodies used

anti-FLAG (M2 clone, mouse) magnetic beads (M8823, Sigma), anti-V5 (clone V5010, mouse) agarose beads (A7345, Sigma) , custom anti-FRQ, anti-FRH and anti-WC1 polyclonal primaries (rabbit) courtesy of Dunlap-Loros labs at Dartmouth, Goat anti-Rabbit polyclonal (Invitrogen, 31460), anti-HA primary (clone 2-2.2.14, mouse) (Invitrogen, 26183), Goat anti-Mouse polyclonal secondary (Invitrogen, 31430), anti-V5 (clone SV5-Pk1, mouse) primary (Invitrogen, 46-1157).

Validation

Manufacturer validations and other published papers using these antibodies are available at applicable vendor websites. Custom antibodies were validated and previously published, with references in our paper. Additionally, we used positive (tagged strains) and negative (untagged strains or gene KO's) controls in our Co-IPs and western blots to ensure antibodies are detecting the appropriate protein target, along with verifying the size of detected protein bands in blots. Different antibodies in different backgrounds (mouse vs. rabbit) were also used to verify antibodies were detecting the appropriate protein(s).

Plants

Seed stocks

N/A, though some fungal strains were obtained from the Fungal Genetics Stock Center, and strains generated in this study will also be deposited there.

Novel plant genotypes

N/A

Authentication

N/A
